# Cognitive and Neural Effects of a Brief Nonsymbolic Approximate Arithmetic Training in Healthy First Grade Children

**DOI:** 10.3389/fnint.2018.00028

**Published:** 2018-07-17

**Authors:** Camilo Gouet, César A. Gutiérrez Silva, Bruno Guedes, Marcela Peña

**Affiliations:** ^1^Cognitive Neuroscience Laboratory, Pontificia Universidad Católica de Chile, Santiago, Chile; ^2^Department of Neuroscience, King’s College of London, London, United Kingdom

**Keywords:** approximate number system, cognitive gain, brain plasticity, first grader, training

## Abstract

Recent studies with children and adults have shown that the abilities of the Approximate Number System (ANS), which operates from early infancy and allows estimating the number of elements in a set without symbols, are trainable and transferable to symbolic arithmetic abilities. Here we investigated the brain correlates of these training effects, which are currently unknown. We trained two Groups of first grade children, one in performing nonsymbolic additions with dot arrays (Addition-Group) and another one in performing color comparisons of the same arrays (Color-Group). The training program was computerized, throughout seven sessions and had a pretest-posttest design. To evaluate cognitive gains, we measured math skills before and after the training. To measure the brain changes, we used electroencephalogram (EEG) recordings in the first and the last training sessions. We explored the changes in N1 and P2p, which are two electrophysiological components sensitive to nonsymbolic numeric computations. A passive Control-Group receiving no intervention also had their math skills evaluated. We found that the two training Groups had similarly gain in math skills, suggesting no specific transfer of the nonsymbolic addition training to math skills at the behavioral level. In contrast, at the brain level, we found that only in the Addition-Group the P2p amplitude significantly increased across sessions. Notably, the gain in P2p amplitude positively correlated with the gain in math abilities. Together, our results showed that first graders rapidly gained in math skills by different interventions. However, number-related brain networks seem to be particularly sensitive to nonsymbolic arithmetic training.

## Introduction

Mathematical skills are crucial to succeed in modern societies (e.g., Duncan et al., 2007; Ritchie and Bates, 2013; Schley and Peters, 2014). Converging evidence suggests that these skills are partially built from an evolutionarily ancient nonsymbolic number sense, supported by the so called Approximate Number System (thereafter ANS). ANS allows us to make quantitative estimations without symbols (Dehaene, 2005; Hubbard et al., 2008; Libertus et al., 2013). Indeed, ANS capacities observed in 14-year-old children positively correlate with children’s past scores on symbolic math tests, including their kindergarten scores (Halberda et al., 2008). Although this and other studies have reported similar associations between ANS capacities and symbolic mathematics (see meta-analysis in Fazio et al., 2014), others have failed to observe such associations (De Smedt et al., 2013) or either have suggested a predominant role of domain-general functions in explaining these associations (Gilmore et al., 2013). Recent training studies have addressed these issues, providing evidence for causal links between nonsymbolic and symbolic math abilities (Park and Brannon, 2013; Hyde et al., 2014). Here we investigated the brain correlates of these training effects.

In a series of studies, Park and Brannon (2013, 2014) showed transfer effects from nonsymbolic arithmetic training to symbolic arithmetic in adults. They observed that subjects trained in solving approximate additions or subtractions of dot arrays, under conditions that prevent them from counting, outperformed subjects trained in other cognitive tasks in a subsequent symbolic arithmetic test. More recently, Hyde et al. (2014) reported similar results with children, showing that their performance in a symbolic exact arithmetic test enhanced significantly more after practicing nonsymbolic addition than after training other non-numeric tasks (see also Wang et al., 2016). Most importantly, these effects cannot be explained by exercising domain-general functions such as inhibitory control or attention, nor can be accounted for by expectation or placebo effects (Dillon et al., 2015).

While these studies provide converging behavioral evidence of transfer effects from ANS to symbolic math skills, the brain mechanisms underlying these effects remain unknown. Indeed, although several studies have explored the brain basis of numerical cognition throughout development, pointing to the Intraparietal Sulcus (IPS) as a key brain region (e.g., Nieder and Dehaene, 2009), it is still unknown whether the malleability of the ANS has detectable brain correlates, and whether those changes may relate to transfer effects on symbolic numerical abilities.

Recent electrophysiological studies have identified two main brain signatures of the ANS, i.e., N1 and P2p, both observed over parietal electrodes (Dehaene, 1996; Temple and Posner, 1998; Libertus et al., 2007; Hyde and Spelke, 2009; Rubinsten et al., 2013). The N1, is the first negative component peaking around 150 ms poststimulus, and the P2p, the second positivity extending from nearly 200 ms to 450 ms poststimulus. N1 and P2p would represent the early and late steps of the number-related processing. P2p is thought to arise from the recruitment of parietal networks involved in numerical processing (Piazza et al., 2004) and the increase of its amplitude would reveal the effort to discriminate between arrays of elements, which is a function of its ratio i.e., the Weber law, (Dehaene, 1996; Temple and Posner, 1998; Libertus et al., 2007; Liu et al., 2018). Some studies have reported that P2p amplitude is influenced by the evaluation of the perceptual visual features of dots arrays (e.g., Gebuis and Reynvoet, 2013). However, recent studies have shown that the simple exposure to large numeric visual stimuli, such as dots arrays, did not associate with increasing of the P2p amplitude, unless the participant attention was committed to manipulate the numeric aspects of those stimuli (Szücs and Soltész, 2008; Soltész et al., 2010; Soltész and Szücs, 2014). These studies supported the idea that, the changes in P2p amplitude reflect important aspects of the numeric processing, besides the perception of the numeric stimuli. Concerning the N1 component, children and adults’ studies have reported that its amplitude depends on the changes in the absolute number of elements in small arrays, but not on their ratio (Libertus et al., 2007; Hyde and Spelke, 2009, 2011). Since we used large number dots arrays in this study, we focused our brain non-null hypothesis on the P2p component.

Although the P2p component is recognized as an index of ANS activity, it is unclear whether the malleability of the ANS through intensive training experiences may correlate with changes in this brain signature. More importantly, despite the fact that this component has also been reported as being the responsible for underpinning the processing of symbolic numbers (Dehaene, 1996), just a few studies have explored its direct relationship with paper-and-pencil symbolic math skills either with children or adults (but see Hyde et al., 2016). To examine these issues, we implemented a computerized game-based training program for healthy first-grade children. We used a pretest-posttest design comprised of three experimental Groups: the first one was trained in solving nonsymbolic approximate additions with dot arrays (Addition-Group); the second one was trained in comparing the color of the same dots arrays (Color-Group), which was comprised of an active control group; and the third passive Control-Group (Control-Group) did not receive any training. Before and after the training, children of all Groups completed a battery of math and verbal skills tests. The training program consisted of seven sessions, one per day, mostly applied in consecutive days at their school. Children in the Addition and Color Groups completed sessions 1 and 7 in the laboratory, and their scalp electrical activity was recorded by using an electroencephalogram (EEG).

At the behavioral level, our expectations were to show that: (a) nonsymbolic approximate arithmetic would be trainable in children (i.e., the Addition-Group would improve at the training task); and (b) such training would transfer to symbolic arithmetic math skills. We expected that the Color-Group would also improve in their training task, but we did not expect transfer effects to symbolic arithmetic tests, being consistent with previous reports with children (e.g., Hyde et al., 2014). It is worth noting that we used a larger battery of math tests than those typically used in similar studies, which commonly have assessed symbolic arithmetic skills (e.g., Park and Brannon, 2013, 2014; Hyde et al., 2014; but see; Khanum et al., 2016). Thus, the aforementioned predictions on transfer effects were held for the symbolic arithmetic tests we implemented (i.e., Mental Operations and Written arithmetic). But in complement, we also evaluated children in the Number Line tasks and in a Numeration test. The Number Line task measures children’s ability to associate spatial and numerical magnitudes (Siegler and Booth, 2004). Based on previous evidence showing positive associations between performance in this task and performance in a nonsymbolic number comparison task (Fazio et al., 2014), we predicted improvements in the Number Line task in the Addition-Group only, albeit these effects had been reported as weaker than those for the arithmetic tests (see Khanum et al., 2016). We also expected some benefits for the Color-Group in this task, since solving the color comparison task required them to exercise the comparison of a continuous magnitude (i.e., more or less similar in color intensity, with regards to the color of the whole dots array), which would benefit the processing of spatial magnitudes (see Khanum et al., 2016). Regarding the Numeration test, it measured children’s basic conceptual knowledge of numbers such as counting. We did not have a pre-conceived expectation regarding this test, although it would not be particularly improved by the nonsymbolic arithmetic training. Additionally, we assessed children’s acuity for nonsymbolic number comparison (Halberda et al., 2008, i.e., Panamath). Here we expected improvements for the Addition-Group and not for the Color-Group, although they may not be strong (see Hyde et al., 2014). Finally, vocabulary skills were evaluated to test the specificity of our intervention in the realm of math. We expected that all Groups would equally gain in this task.

At the brain level, we expected that: (a) the Addition-Group would show significantly greater changes in the P2p component (between session 1 and 7) than the Color-Group, revealing specific brain changes underpinning the improvements in nonsymbolic arithmetic computations; and (b) the gains in at least one symbolic arithmetic skill would correlate with changes in this ERP component. This was expected under the assumption that the P2p component reflects the brain activity of a parietal network involved in processing both symbolic and nonsymbolic numerical magnitudes (e.g., Piazza et al., 2004). If our predictions were correct, then we would have identified valuable neurocognitive evidence linking intuitive and formal math abilities in young children.

## Materials and Methods

In this study, we explored whether a brief computerized intervention, comprised of seven sessions of nonsymbolic approximated addition: (a) improved its accuracy, demonstrating that it is trainable in first-grade children; (b) was associated with gain in symbolic math performance, demonstrating transfer effects; (c) was associated with adaptations in the brain response (i.e., the amplitude of the P2p); and (d) revealed significant correlations between the gains in training performance and/or math skills with the gain in P2p component.

### Participants

Eighty-two healthy first-graders were recruited for participating in the study. One participant was excluded from the study because she did not complete the training protocol. Fifty-nine children were pseudo-randomly assigned either to the Addition or the Color Groups. Groups were counterbalanced by taking into account their scores in a series of cognitive assessments obtained before the beginning of the intervention. We assured thus that the participants of both Groups did not already differ in cognitive abilities prior to the intervention. In the Addition-Group (*n* = 30, 15 females, mean age: 6.03 years, range: 6–7 years), participants were instructed to mentally add two sets of dots, while in the Color-Group (*n* = 28, 15 females, mean age: 6.05 years, range: 6–7 years), children had to compare the color intensity of a target dot array, with the color of one of the two arrays previously seen (see below). By using a 64-channels EEG, EGI Inc. system, 23 children from the Addition-Group and 21 from the Color-Group had their brain activity recorded during the first and last training sessions (i.e., 1st and 7th sessions). Two children from the Addition-Group and seven from the Color-Group were excluded from statistical analyses of the EEG data because their recordings had less than 15 free-of-artifact trials, thus 21 and 14 children remained for the analyses of brain signals from each Group respectively. To estimate the influence of other factors in math skills such as school instruction during the intervention period, we also evaluated a passive Control-Group (*n* = 33, 15 females, mean age: 6.06 years, range: 6–7 years) who did not receive any training. We applied to this Group the same cognitive assessment battery used for the other two Groups, in two occasions, separated by the time equivalent to the average duration of the training in the intervened groups. All children were evaluated during the first 3 months of their school year. We estimated the sample size in *n* ~30 per Group, taking into consideration the sample size reported by previous studies with adults and children (Park and Brannon, 2013, 2014; Hyde et al., 2014). The study was aproved by the Ethical Committee of the School of Psychology of the Pontificia Universidad Católica de Chile. Children gave verbal assent and parents signed a written consent form to allow each child to participate in the study.

### Training Tasks

**Figure 1:**
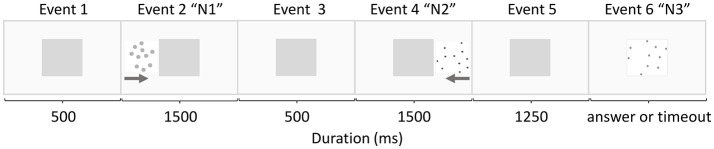
Schematic distribution of the events in each trial. In event 2 and 4, the gray arrows represent the movement of the dots behind the central square from the left and the right sides, respectively.

Participants were trained in Groups of 10–12 students in a quiet room at their school, throughout seven sessions, mostly in consecutive days. Each child practiced on a personal computer. Before starting a session, a Group of examiners registered each child’s personal information and reminded them the instructions of the game. The trial structure was the same in both training tasks, and it was similar to the one implemented by Hyde et al. (2014). Each trial was comprised of six visual events that are illustrated in Figure 1. Event 1: a central, non-transparent yellow square appeared in the middle of the screen for 500 ms; Event 2: an array of dots (N1) appeared on the left side of the central square and moved behind it in 1500 ms; Event 3: the central square remained alone for 500 ms; Event 4: a second array (N2) appeared on the right side of the central square and moved behind it in 1500 ms; Events 5: the central square remained alone for 1250 ms; and in Event 6 the central square disappeared, uncovering a third array of dots (N3), which was presented until a response was provided, or a timeout equal to 3000 ms was reached. All dots array images were generated by using the Matlab script provided by Dehaene et al. unpublished manuscript.

Children from the Addition-Group had to decide if N3 had more or less dots than the sum of N1 and N2. If they thought this sum had less dots than N3, they would press a key on the left side of the keyboard, or if they thought this sum had more dots than N3, they would press a key on the right side. The short time of arrays presentations prevented children from counting the dots, thus motivating the use of ANS estimation to solve the task. The arrays for N1, N2 and N3 were presented in the same color but with different levels of saturation (which was perceived as difference in color intensity). In order to obtain as many similar trials for ERP analyses as possible, we selected only two levels of numeric difficulty for this task. Nonsymbolic addition task difficulty was thus controlled by varying the ratio between the actual sum of the addends (N1 and N2) and the number of dots displayed in the third array (N3). Half of the trials were “Easy” with a ratio of 0.57, and half of them were “Hard” with a ratio of 0.71. The number of dots in each array ranged from 7 to 43 (17 dots on average). The values of the actual sums ranged from 16 to 56 dots (36 dots on average). To prevent children from using non-numerical parameters that co-vary with numerosity when solving the task, half of the trials presented dots equated in the occupied area, and the other half presented dots with the same individual area (as typically done in nonsymbolic number studies, e.g., Hyde and Spelke, 2009; Fazio et al., 2014).

Children from the Color-Group were exposed to identical stimuli and experimental protocol as the Addition-Group, but in this case, they were asked to compare the color of the dots arrays and decide if N3 was more similar in color to N1 or N2. The idea of the sum of the dots was not even suggested to the children of this Group. The correct response consisted in selecting the closest arrays in color. Indeed, the arrays differed in color intensity in such a way that the N3 array was closer in color intensity to either the N1 or the N2 array. The closer distance was 0.51 points in the RGB color model saturation scale, while the farthest distance was 0.71 points in the same scale. In half of the trials, the N3’s color saturation was closer to the N1 array and in the other half of the trials to the N2 array (randomly ordered within each session).

Previous studies have demonstrated that although nonsymbolic addition and color comparison tasks involve the processing of magnitudes, they are essentially different (Gilmore et al., 2010). Indeed, while the nonsymbolic approximate addition is numeric in nature, the color comparison required for the processing of a continuous magnitude, even when the stimuli are composed of arrays of discrete elements. Moreover, to succeed in our addition task, children should somehow neglect the perceptual properties of the dots arrays and focus on the arithmetic manipulation of their numeric properties. In contrast, to succeed in the color task, children should focus on the perceptual properties of the dots arrays, i.e., color intensity, disregarding the numeric ones. Furthermore, we believed that our tasks provided children with similar opportunities to train their general cognitive abilities, such as working memory, although those improvements would entail different aspects of this ability, depending on the goal of the task. Indeed, to solve the tasks children would have to keep in their mind the numeric or the perceptual properties of N1 and N2 arrays until N3 (the target) appeared, which happens 3 s after the onset of the trial. While the color task would rely on holding the visual information available to make the perceptual matching (being closest to a short-term memory process), the addition task would require the manipulation of the stored information, in the domain of arithmetic. In sum, the tasks we designed here aimed to capture as many differences as possible in the cognitive and brain adaptations that young children developed when they manipulate stimuli physically identical, in the domain of approximate mental arithmetic, i.e., approximate addition task; vs. when they manipulate them as a nonnumerical comparison i.e., color comparison task.

Nevertheless, in order to avoid any eventual influence of the perceptual factors of our visual stimuli on any aspects of our results, in further analyses we compared the data of the set of Easy and Hard trials of the Addition-Group with the identical set of trials of the Color-Group. We also called them Easy and Hard trials in the Color-Group, although they did not differ in their level of difficulty.

Trials were presented in a random order. The 1st and 7th sessions (were coupled with EEG recordings and conducted in the laboratory) included 80 trials, while the other sessions (conducted in the school) were comprised of 40 trials each. School sessions lasted from 10 min to 15 min each while EEG sessions lasted 40–45 min, including technical issues. To keep children engaged in the training, we varied the color of the dots across sessions, with blue, green, red, magenta and cyan for school sessions, and gray for the two EEG sessions. All children were requested to provide their answer as fast and accurate as possible.

### Cognitive Assessments

Cognitive assessments were comprised of the tasks described below. They were individually applied to each child by psychologists, who were blind to the objectives of this research, from 1 weeks to 2 weeks before the intervention began and from 1 weeks to 2 weeks after the intervention ended.

#### Symbolic Math

We used three subtests of the Key-Math battery (Connolly, 2007). The “Mental Computation and Estimation” (thereafter Mental Operations) and “Addition and Subtraction” (thereafter Written Arithmetic) subtests, which were critical to estimate the symbolic arithmetic knowledge, since they evaluate children’s arithmetic procedural skills. The third subset was “Numeration,” which assessed the children’s conceptual knowledge of numbers and their basic operations (e.g., counting). The internal reliability of these subset for Key Math battery has been reported with a Mean Split-Half Reliability coefficient > 0.9 in first graders.

#### Number Line Task

This task measures children’s spatial representation of numbers (Barth and Paladino, 2011). We used the same 26 digits and the protocol that were previously used by Slusser et al. (2013). In each trial, children were requested to mark with a pencil the position where each digit was supposed to be on a 23 cm long number line, drawn on paper, depicting the number 0 at the beginning and the number 100 at the end, on their left and right extremes of the line, respectively.

#### Nonsymbolic Number Comparison Task

We implemented the task developed by Halberda et al. (2008, i.e., Panamath). During the task, children were presented with a series of pairs of dot arrays and they had to decide which array had more elements. One of the arrays depicted yellow dots and the other one, blue dots. The time presentation of the stimuli prevented the kids from counting. We presented approximately 100 trails per children and they followed the same protocol as the one presented in Fazio et al. (2014).

#### Vocabulary

We applied the TEVI-R test (Echeverría et al., 2002), which was used to evaluate comprehensive vocabulary. In each trial, children heard a spoken word and had to match it with the corresponding image from a slide containing four different images. It is analogous to the Peabody Picture Vocabulary Test (Dunn and Dunn, 1981). The reported internal reliability of this test has a Cronbach alpha > 0.85 for first graders.

The cognitive assessments were administered in a quiet room at their school, in one session with a brief break between tests. The testing order was always Symbolic Mathematic first, followed by Vocabulary or Number Line. Evaluation session lasted ~50 min per child. The nonsymbolic number comparison task was applied on a separate day in a specially adapted room with computers. The Symbolic Math and Vocabulary tests were applied before the training was started, which allowed us to equate the Groups in these abilities prior to performing the intervention. We specifically computed a composite measure by pondering each symbolic math subtest score with 0.25 and vocabulary test with 0.25, being math notion pre-eminent (75%) over vocabulary (25%). The time between pre- and post-training evaluation was on average 54.4 days (range: 42–63 days).

### EEG Data Acquisition

Scalp voltages were continuously recorded by using a 64-channel EEG system (EGI Inc., Eugene, OR, USA), digitized at a sampling rate of 500 Hz. EEG was first filtered (bandpass filter = 0.5–20 Hz) and then segmented into 1.4 s long epochs including 200 ms before to N3’s onset. Channels contaminated by eye or motion artifacts (i.e., voltage fluctuations exceeding 100 μV or transients exceeding 100 μV) were automatically rejected and trials with more than 10% of bad channels were excluded. Maximal impedance was 40 kΩ. Non-rejected trials were averaged, baseline corrected across the 200 ms before the N3’s onset and transformed into an average reference. The number of the trials kept for further analyses ranged from 14 to 28 for the Easy trials and from 15 to 27 for the hard ones. In the procedures described here, we used EEGLAB (Delorme and Makeig, 2004), an open-source Matlab toolbox for EEG analysis.

### Data Analysis and Statistics

#### Training Data

To quantify the gains across sessions, we first computed the accuracy (i.e., percentage of correct responses) and reaction time (RT) of each child from each Group in every session. We then submitted the values for those variables to a reliability test. Cronbach’s alpha > 0.7 across sessions was used to consider that accuracy and RT consistently measured similar cognitive capacities in each Group. After confirming that the tasks were reliable, we submitted the accuracy and RT to a series of statistical comparisons.

Accuracy scores were compared against chance (50%) by using a one-sample *t*-test (two tails, alpha 0.05) to evaluate if children succeeded in the tasks in every session, and for each Type of Trial. Just to remind, the classification of Easy and Hard trials for the Color-Group corresponded to the trials that were physically identical to the Easy and Hard trials in the Addition-Group. We also submitted the mean accuracy and mean RT for the correct and wrong responses to a series of two independent samples *t*-test (two tails, alpha 0.05) so as to identify differences between Groups in each Type of Trial and session.

To measure the progress in the mean accuracy across the seven sessions of each child, we computed the slope in accuracy across the sessions in each Type of Trial. Then, we submitted the slopes value to a comparison between Groups and against zero (i.e., no linear change in mean accuracy across training sessions). Moreover, we complemented this analysis by comparing, in each Group, the mean accuracy in session 1 against the mean accuracy in the rest of the sessions averaged together, for each Type of Trial.

#### Gains in Cognitive Assessments

We first confirmed that Groups did not differ in math and vocabulary test scores before the training was started, by submitting the pre-training raw scores of the children of each Group in each task, to a series of two independent samples *t*-test (two tails, alpha 0.05), one per task. We then verified the internal consistency of the Key Math subtests by measuring math skills with the children from the current study and computing the reliability for the raw scores in numeracy, mental operations and written arithmetic, for both pre- and post-training evaluations in each Group. Cronbach’s alpha > 0.7 was considered reliable.

After demonstrating internal consistency for math tasks, we proceed to analyze their gains. To measure the effects of the training in cognitive tasks, we standardized the gain scores by subtracting each participant’s pre-training score from the post-training one and dividing it by the standard deviation of the pre-training scores across all participants, as it has been applied in previous ANS training studies (Park and Brannon, 2013, 2014). To compute the standardized gain scores in symbolic math and vocabulary, we used the raw scores (ceiling—errors). For the number line task, we used each child’s Percentage of Absolute Error (PAE) averaged across all digits, computed as: 100 * (estimated position − true position)/(length of the line). Results at *p* < 0.05 were considered statistically significant for this test.

For each cognitive task, we assessed the transfer by testing whether each Group significantly improved after the training (i.e., obtained gains scores above zero), and by testing if there were differences in the observed gains between Groups. We used planned *t*-tests for our *a-priori* hypotheses that were inspired by the literature and that were presented in the Introduction. We corrected the significance level of alpha (0.05) using Bonferroni criteria when testing either a small set of exploratory *post hoc* hypotheses or a full range of *post hoc* pairwise comparisons. These will be indicated in each case. Finally, to check that the differences in the cognitive assessments were not due to differences that the children already had before the intervention, we submitted the gains in each task to an ANCOVA analysis controlling by the scores observed before the training started.

#### EEG Data Analysis

The EEG analysis considered exclusively the trials with correct responses, because only three children from the Color-Group and five from the Addition-Group had more than 15 non-rejected wrong trials, partially due to the fact that those children made more movements when they provided wrong responses. We started the analysis by using unbiased statistics, to identify spatiotemporal clusters over which we should run the statistical comparisons to test our hypotheses. We applied cluster-based permutation analysis (Maris and Oostenveld, 2007), a Fieldtrip function (Oostenveld et al., 2011) integrated into Brainstorm (Tadel et al., 2011), both open-source applications for brain recordings analyses. The spatiotemporal clusters would thus indicate us where and when the electrophysiological activity of the brain significantly differed between Groups, training sessions and/or Type of Trial.

Initially, we looked for main effects of Group or Session without any *a-priori* assumption regarding the electrodes and/or time windows where we should have looked for statistical differences, avoiding double dipping effects. We thus submitted the EEG data from each child of each Group (Addition vs. Color), regardless of the Type of Trial (Easy and Hard) and Session (1st and 7th), to a cluster-based permutation analysis, with 1000 iterations; threshold of *p* = 0.05, FDR corrected, over the time window from 0 ms to 1200 ms after the N3’ onset. Previous studies have shown that during this time window, the symbolic and nonsymbolic processing of number, influences the amplitude of the P2p component in adults (e.g., Dehaene, 1996; Temple and Posner, 1998). We then proceeded in similar fashion to explore the eventual main effect of Session by submitting the data from each child per session (1st vs. 7th) to a cluster-based permutation analysis, regardless of the Group (Addition and Color) and Type of Trial (Easy and Hard).

After identifying the spatiotemporal clusters for significant differences, we computed the average of voltage amplitudes over the cluster, and the latency of the peak amplitude of those averages, for each child of each Group (Addition and Color) in each Type of Trial (Easy and Hard) and in each Session (1st and 7th). We then submitted those values of amplitude and latency to an ANOVA with Session (1st and 7th) and Type of Trial (Easy and Hard) as within-subject factors, and Group (Addition and Color) as between-subject factor, with FDR correction for multiple comparisons and noting partial eta squared ($\eta_{p}^{2}$ ) effect sizes for all significant effects and interactions.

#### Correlations

We submitted each of our gain data from cognitive assessments, training and EEG recordings to correlation and regression analyses, in an attempt to identify dependency relationship between them.

## Results

### Training Performance

As we found significant effects only in accuracy, not in RT in the training tasks, therefore we focused on this measure. The first analysis showed that the mean accuracy across all trials was significantly greater than chance (50%) for every session in both Groups (*p* < 0.031 for each comparison), indicating that children succeeded in solving the tasks across sessions.

In the Addition-Group, we also found that the accuracy was higher for the Easy than for the Hard trials in all sessions (*p* < 0.05 for each comparison), except in session 2 for the Hard trials (*p* = 0.301), confirming the two levels of difficulty for this task.

**Figure 2:**
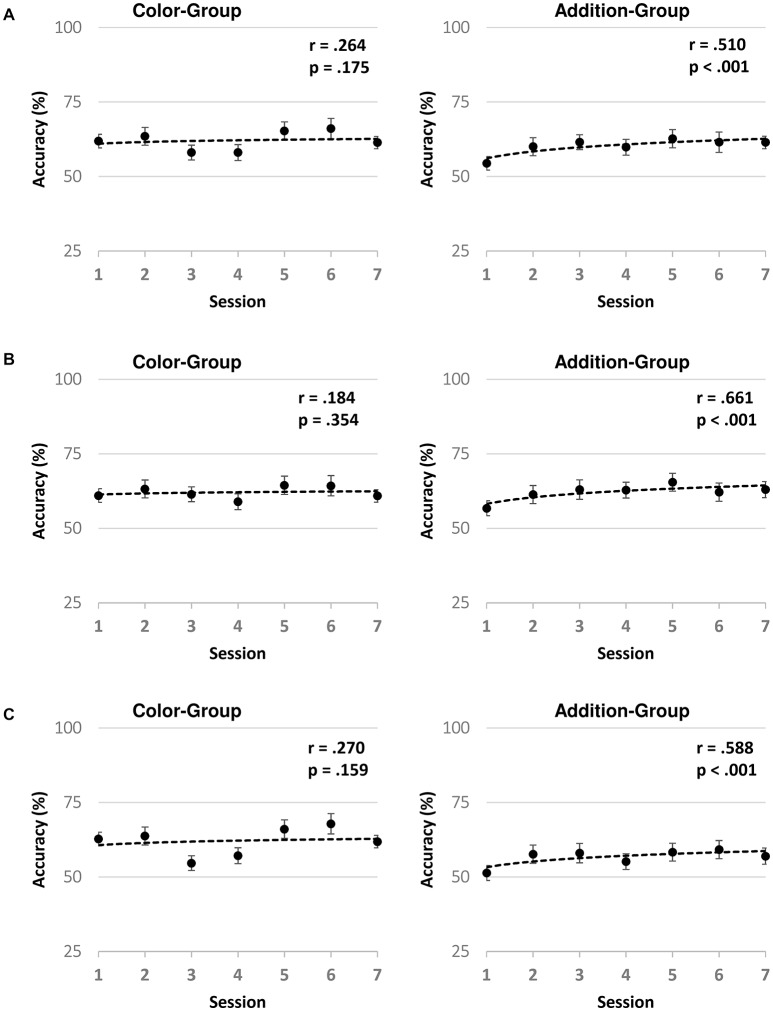
Logarithmic fitting for the mean accuracy across sessions per Group and Type of Trial. Fifty percent accuracy indicates performance level expected by chance. In **(A)** we illustrate the data from all Types of Trials together, while in **(B,C)** those from the Easy and Hard trials. Dots correspond to the mean of the Group in each session, and error bars indicate the standard error for each mean. The logarithmic fitting curve for the mean of the Group across sessions is plotted with a black dotted line. The Pearson’s correlation coefficients and their corresponding *p*-value is shown for each plot.

Next, we evaluated the progress in accuracy across training in each Group by regressing accuracy on training sessions, in each child. When testing mean regression slopes against zero (i.e., no training progress) for all trials (i.e., collapsing Easy and Hard trials) we found significant improvements only for the Addition-Group (*t*_(59)_ = 3.170, *p* = 0.002, Cohen’s *d* = 0.260) with no significant changes for Color-Group (*t*_(55)_ = 0.394, *p* = 0.789). We observed similar results when we separately analyzed the slopes by Type of Trial, observing improvements in accuracy for the Easy and Hard trials (*t*_(29)_ = 2.312, *p* = 0.028, Cohen’s *d* = 0.422 and *t*_(29)_ = 2.162, *p* = 0.038, Cohen’s *d* = 0.395, respectively). Similar results were found when we submitted the mean accuracy of each Group against Session to a correlation analysis. We found a positive correlation of the mean accuracy only for the Addition-Group for the Easy (Pearson’s correlation coefficient = 0.661, *p* < 0.001) and Hard trials (Pearson’s correlation coefficient = 0.588, *p* < 0.001) and for all trials collapsed together (Pearson’s correlation coefficient = 0.510, *p* < 0.001), see Figure 2. In contrast, the mean accuracy of the Color-Group did not show any significant correlations for similar comparisons (*p* > 0.150 for each comparison). Finally, consistent with those results, we also observed that only in the Addition-Group, the mean accuracy in the 1st session was significantly smaller than the mean of the accuracy of the rest of the sessions collapsing both, Easy and Hard trials (*t*_(29)_ = 2.048, *p* = 0.028, Cohen’s *d* = 0.373 and *t*_(27)_ = 2.052, *p* = 0.010, Cohen’s *d* = 0.374, respectively).

Thus, our training results showed that both trained Groups performed significantly above chance in all training sessions, suggesting that both of them understood and were committed to the training games. However, only the Addition-Group’s task appeared to be trainable by the intervention. Indeed, only the Addition-Group significantly improved in performance across training sessions, while the Color-Group exhibited large fluctuations in accuracy across sessions, converging into a flat gaining slope. The fact that the participants from the Color-Group have had high performance in the early sessions of the training cannot completely explain the changes in accuracy across sessions, since we may have expected that their accuracy at least remained near the performance observed in the first session.

#### Gains in Cognitive Assessments

**Figure 3:**
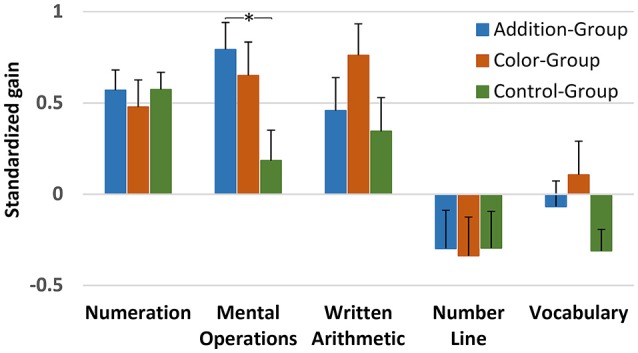
Standardized gain scores in cognitive tasks per Group. Error bars indicate the standard error of the mean. All the Groups significantly gain when they are compared against zero-gain (*p* < 0.05). The asterisk indicates the only significant difference observed between groups, i.e., between Addition and Control Group at *p* = 0.017.

The pre- and post-scores in each cognitive task and in each Group are illustrated in Table 1, and Figure 3 summarizes the gains in cognitive assessments. All Groups showed significant gain in more than one cognitive task when we compared them against zero-gain. Consistent with our predictions, the Addition-Group showed significant improvement in both symbolic tests i.e., Written Arithmetic (Mean ± SD = 0.458 ± 0.983; *t*_(29)_ = 2.530, *p* = 0.017, Cohen’s *d* = 0.832) and Mental Operations (Mean ± SD = 0.793 ± 0.924; *t*_(29)_ = 5.323, *p* < 0.001, Cohen’s *d* = 0.468). However, a similar pattern of gains was observed in Color-Group (Mean ± SD = 0.793 ± 0.924; *t*_(27)_ = 5.323, *p* < 0.001, Cohen’s *d* = 0.468 for Written Arithmetic; and Mean ± SD = 0.650 ± 1.119; *t*_(27)_ = 3.540, *p* = 0.001, Cohen’s *d* = 0.779 for Mental Operations), indicating that the gains in these tasks were not specific to the training of nonsymbolic arithmetic.

**Table 1:** The mean score ± the standard deviation in each cognitive task for pre- and post-training evaluation per Group.

	Color-Group		Addition-Group		Control-Group	
TASK	Pre	Post	Pre	Post	Pre	Post
Numeration	8.2 ± 3.0	9.5 ± 3.5	8.4 ± 3.2	9.9 ± 3.3	7.4 ± 2.0	8.8 ± 2.0
Mental operation	4.0 ± 1.8	5.4 ± 2.4	3.8 ± 2.7	5.4 ± 2.5	4.5 ± 1.2	4.7 ± 1.5
Written arithmetic	4.2 ± 2.5	5.8 ± 2.3	4.5 ± 2.3	5.5 ± 3.0	4.9 ± 1.6	5.7 ± 2.1
Number line	18.3 ± 8.6	16.3 ± 8.3	20.6 ± 8.4	18.0 ± 9.2	22.5 ± 8.5	19.9 ± 7.8
Vocabulary	44.8 ± 7.9	45.6 ± 5.7	44.6 ± 9.1	44.1 ± 6.0	46.7 ± 5.3	45.3 ± 8.3

The gain in symbolic math for the Color-Group could be explained by improvements in the continuous magnitude processing as well as in domain-general cognitive skills such as working memory that have been previously reported as causal agents for improvements in numeric processing (e.g., Kolkman et al., 2013; Xenidou-Dervou et al., 2013). However, further studies are necessary to explore those possibilities.

The results on the Number Line task revealed significant gains in the Addition-Group (Mean ± SD = −0.297 ± 0.784; *t*_(29)_ = −2.124, *p* = 0.042, Cohen’s *d* = 0.382) but not in the Color-Group, showing that this spatial task did not benefit from exercising the manipulation of color as a continuous magnitude. Although the Addition-Group showed a marginal advantage when compared to the Color-Group in this task, this gain was not exclusive for this Group considering that we also found it in the Control-Group (Mean ± SD = −0.295 ± 0.671; *t*_(32)_ = −2.543, *p* = 0.016, Cohen’s *d* = 0.444), suggesting that other than the training program could be at the basis of the gains in the Number Line task.

In the Numeration test, we observed equally significant gains across all experimental Groups (Control-Group: Mean ± SD = 0.573 ± 0.580; *t*_(32)_ = 6.173, *p* < 0.001, Cohen’s *d* = 1.074; Addition-Group: Mean ± SD = 0.569 ± 0.679; *t*_(29)_ = 5.073, *p* < 0.001, Cohen’s *d* = 0.926; and Color-Group: Mean ± SD = 0.477 ± 0.871; *t*_(27)_ = 3.204, *p* = 0.003, Cohen’s *d* = 0.605), suggesting that other than the training factors could be at the basis of the gains in this task.

Also contrary to our expectations, no gains were observed in ANS acuity (i.e., Weber fraction) in any Group (data not shown), although previous studies have shown similar lack with school children (Hyde et al., 2014). This lack of gain could be associated with the briefness of the current training or to the fact that our stimuli conveyed just a few (i.e., two) numeric ratio, which could be not variable enough to train and benefit ANS acuity.

Finally, as expected, in none of the Groups did we observe improvements in Vocabulary, suggesting that the gains in symbolic representations were restricted to the realm of math.

Despite the fact that we did not find any cognitive task in which only the Addition-Group showed significant gain, the comparison between Groups revealed interesting results. Indeed, the comparison of the standardized gains between Groups showed a significant main effect of Group only in Mental Operations (*F*_(2,89)_ = 4.274, *p* = 0.017; $\eta_{p}^{2}$ = 0.089), with the Addition-Group showing a significantly greater gain than the Control-Group in this task (*F*_(1,62)_ = 7.340, *p* = 0.009; $\eta_{p}^{2}$ = 0.107), with no differences between the Addition and Color-Group (*p* = 0.714) or between Color and Control-Group (*p* = 0.213). This result could not be explained by differences between Groups that already existed before the training had started. Indeed, when we submitted the gains in cognitive assessments to an analysis of covariance (ANCOVA), with pretest scores in each task entered as covariates, we confirmed the significant effect of Group on Mental Operations (*F*_(2,90)_ = 5.434, *p* = 0.023, $\eta_{p}^{2}$ = 0.083) with a negligible Group × Mental Operations pre- training scores interaction (*F*_(2,90)_ = 1.687, *p* = 0.191; $\eta_{p}^{2}$ = 0.038). Similar analyses did not show significant differences in Written Arithmetic, Number Line, Numeration or Vocabulary pretraining scores (*p* > 0.190 in each comparison); and no significant interactions of those scores with Group (*p* > 0.206 in each comparison). Bonferroni correction was applied to each multiple comparison reported in this paragraph.

Taken together, cognitive assessments results suggest a slightly significant transfer effects from the nonsymbolic addition training to symbolic arithmetic skills, particularly to the Mental Operation task. However, the lack of difference between the Addition and the Color-Groups suggests that this effect was not specific to the Addition-Group. The improvements seen in the Color-Group (although nonsignificantly different from the time Control-Group) were contrary to our predictions and could be related to the engagement of continuous magnitude processing, test re-test effects, or to the training of the general abilities such as working memory, which is required to solve color-matching tasks (e.g., Geary, 2011; Kolkman et al., 2013). Although this null result reflected that both Groups behaved and similarly benefitted from a training program, the underpinning brain adaptations associated with those gains might differ between them. The brain evidence that we will present next can help shed light on this issue.

### ERP Results

As in previous analyses, we compared the data from the Easy and the Hard trials of the Addition, with the identical trials of the Color-Group, in order to avoid that our P2p amplitude analyses were influenced by perceptual factors (e.g., Gebuis and Reynvoet, 2013; Liu et al., 2018).

**Figure 4:**
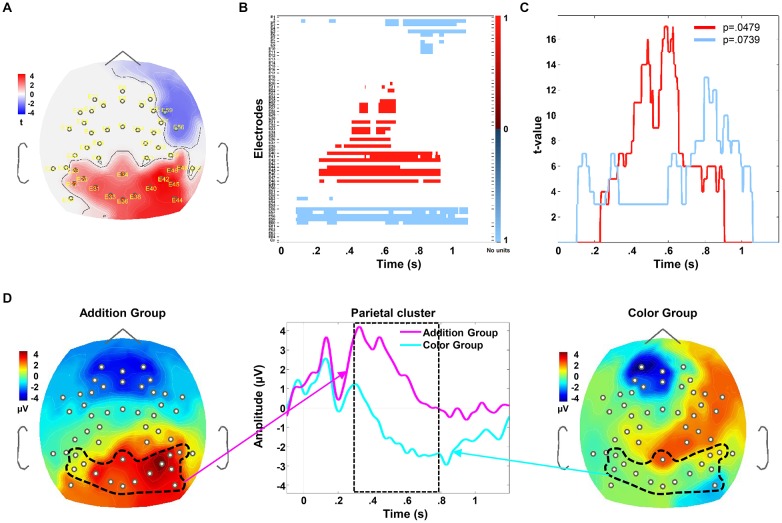
Spatiotemporal cluster for significant differences between Groups, regardless of Session and Type of Trial. Zero time indicates the N3’s onset. **(A)** Draws the scalp topography of the values of the Student’s *t* over the parietal and right-frontal clusters, averaged from 276 ms to 754 ms after the N3’s onset. In **(A–C)**, the red color indicates a greater amplitude of the ERP response in the Addition than in the Color Group, while the blue color indicates the contrary. **(B)** Plots the variation in time of the *t*-value in each electrode. **(C)** Plots the cluster size, illustrating the variation in time of the *t*-values across all the electrodes of each cluster. The depicted *p*-values indicate the statistical probability for each spatiotemporal cluster that emerged when we compared differences between Groups. We further analyzed only the parietal cluster, with a *p* < 0.05. In **(D)**, the middle figure plots the grand averages of the P2p amplitude for the Addition and Color Groups (in magenta and cyan lines, respectively). These means were computed by averaging the electrodes of the parietal cluster delimited by the black dotted in the voltage map images for the Addition-Group (left voltage map, magenta arrow) and Color-Group (right voltage map, cyano arrow), from 100 before to 1200 ms after N3’s onset. The dashed rectangle indicates the time window when Groups significantly differed in the parietal cluster.

Cluster-based permutation analysis allowed us to identify two spatiotemporal clusters (Figures 4A–C), in which the mean amplitude of the brain response to N3 stimulus significantly differed between Groups (*p* < 0.005, FRD correction) regardless of the sessions and types of trial. The first cluster corresponded to a tendency (*p* = 0.073), observed over the right-frontal side of the skull, expanding from 104 ms to 1058 ms after N3’s onset, and involved 2–6 right-frontal electrodes. This component was similar in the spatial distribution and shape to the contingent negative variation (CNV; Walter et al., 1964), a long lasting frontal negative ERPs related to the engagement of selective visual attention and expectancy processes. Alternatively, it may reflect the recruitment of frontal networks engaged in nonsymbolic numerical processing in children of this age (Cantlon and Brannon, 2006). As previous ERP studies on number representation have not reported a similar component and as we did not have pre-defined hypotheses regarding its behavior, we did not further analyze this trend.

The second cluster extended from 276 ms to 754 ms after the N3’s onset and involved 2–5 left-parietal, 1–2 centro-parietal and 5–7 right-parietal electrodes. This response highly overlapped in time and scalp location to the P2p component previously reported for nonsymbolic number processing in adults (e.g., Dehaene, 1996; Temple and Posner, 1998) and young children (Hyde and Spelke, 2009). We thus thereafter referred to our response as P2p. The permutation procedure removed the components of the brain response that were common to both tasks, indicating where and when we should look for statistical differences between Groups, but did not provide us with specific statistical index about those differences (see e.g., Tadel et al., 2011). Thus, to test our hypothesis, which states that the P2p amplitude would be significantly greater for the Addition-Group than for the Color-Group, we first computed the average of the P2p amplitude over the cluster in each child, regardless of Session and Type of Trial, and submitted those averages to a one-way ANOVA with Group (Addition and Color) as between-subject factor (Lilliefors test showed normality for each Group, *p* > 0.05). The grand average over this cluster in each Group is illustrated in Figure 4D. We found a significant main effect of Group (*F*_(1,39)_ = 13.499, *p* < 0.001, $\eta_{p}^{2}$ = 0.262, Bonferroni corrected), confirming that the P2p amplitude was significantly greater for the Addition than for Color-Group across all sessions and trials. However, to evaluate if this greater amplitude were associated with the training program, we proceeded to look for spatiotemporal clusters associated with P2p changes across sessions.

When we looked for clusters distinguishing the brain response across sessions, we did not find any significant one, suggesting that the amplitude of the P2p response was affected by the group, but not by the training factor alone.

**Figure 5:**
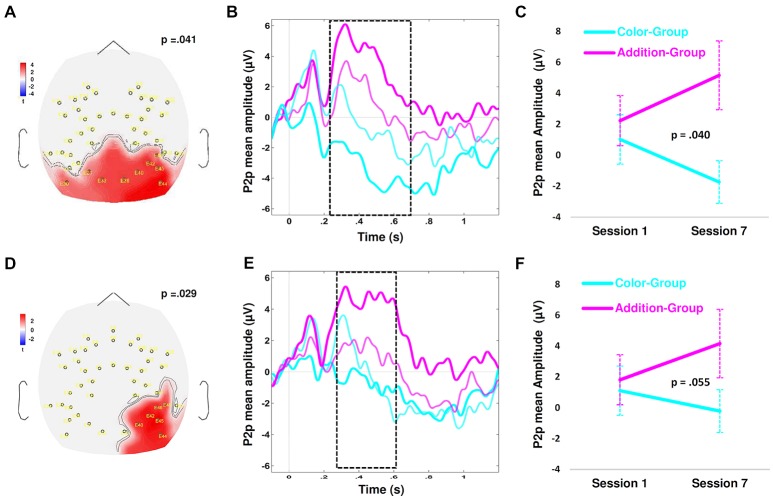
Spatiotemporal cluster for significant differences between Groups per session and Type of Trial. **(A–C)** Illustrate the differences observed between Groups for the Hard trials, across sessions. **(A)** plots the scalp topography of the parietal cluster, indicating the values of Student’s *t* averaged from 256 ms to 754 ms after the N3’s onset, over nine parietal electrodes. The red color indicates the electrodes where P2p amplitude was greater for the Addition than for the Color Group at 7th session. **(B)** draws the time course of the grand averages of the P2p per Group and Session. The magenta and cyan lines correspond to the Addition and Color Groups respectively. The thin and wide lines indicate the averages for session 1 and session 7 respectively. The dashed rectangle indicates the time window when Groups significantly differed in session 7. **(C)** shows the grand average of the P2p amplitude computed over the time and electrodes indicated by the parietal cluster, in the Color and Addition Groups and in session 1 and session 7. The vertical lines show the standard error of the mean. The written p-value corresponds to the p-value of the Group × Session interaction that was obtained when we submitted average of each child to a repeated measure ANOVA (see the text). Panels from **(D–F)** draw the same type of data described for **(A–C)** but now referred to the differences between Groups for the Easy trials across sessions.

However, since we predicted specific significant effects of training for the Addition-Group, we looked for significant interactions between Session and Group, in each Type of Trial, what would have revealed specific differences attributed to the training. To identify such spatiotemporal clusters, in which Groups differed across sessions, to each Type of Trial, we ran a series of cluster-based permutation analysis comparing separately the data from each Group in each Type of Trial, without any *a-priori* assumption about where or when those effects should emerge, avoiding thus double dipping effects. For instance, in one run, we compared the data from the Addition vs. the Color-Group in the first session for the Easy trials; in another run, we compared the data from the Addition and Color-Groups in the 7th session for the Hard trials, and so on. With this procedure, we did not identify any cluster in which the two training Groups differed in session 1 (with *p* > 0.9 for each comparison), indicating that at the beginning of the training, the brain responses of both Groups were similar. Crucially, in session 7, we did identify two parietal clusters in which the mean P2p amplitude for the Addition and the Color groups significantly differed. One cluster appeared when we compared the Hard trials of the Addition-Group against the same trials from the Color-Group. It extended from 256 ms to 754 ms after N3’s onset and involved from 3 to 9 bilateral parietal electrodes (*p* = 0.041; Figure 5A). The other cluster emerged when we compared the Easy trials of the Addition-Group against the corresponding ones from the Color-Group, extending from 246 ms to 558 ms after N3’s onset and involving four right-parietal electrodes (*p* = 0.029; Figure 5D). Both clusters partially overlapped over the right parietal electrodes and were similar in latency, duration and polarity to the cluster described above indicating the spatiotemporal differences between groups. The larger extension of the parietal cluster for the Hard than for the Easy trials in the Addition-Group was congruent with previous results showing that P2p is often modulated by the difficulty of the task and manipulated as increasing the proximity of the number of dots of the arrays that are being compared (Hyde et al., 2016).

After identifying these two clusters when Groups differed across sessions, one for the Easy and one for the Hard trials, we proceeded to look for statistical differences. For each child, we averaged the mean P2p amplitude over each cluster and submitted those averages to two separate repeated measures ANOVAs, one for each Type of Trial, with Session (1st and 7th) as within-subject factor and Group (Addition and Color) as between-subject factor. The grand averages over the two clusters per Group and Type of Trial are illustrated in Figures 5B,E. For the Hard trials we found a significant Group × Session interaction (*F*_(1,33)_ = 4.517, *p* = 0.041, $\eta_{p}^{2}$ = 0.120), due to the P2p mean amplitude was significantly greater in session 7 than in session 1 in the Addition-Group only (*F*_(1,20)_ = 4.824, *p* = 0.040, $\eta_{p}^{2}$ = 0.194), with no significant change in the Color-Group (*p* > 0.45; Figure 5C). For the Easy trials we found a strong tendency for a Group × Session interaction (*F*_(1,33)_ = 3.916, *p* = 0.055, $\eta_{p}^{2}$ = 0.109), due to the P2p mean amplitude was greater in session 7 than in session 1 in the Addition-Group (*F*_(1,20)_ = 4.165, *p* = 0.055, $\eta_{p}^{2}$ = 0.172) with no significant change in the Color-Group (*p* > 0.5; Figure 5F). Greenhouse-Geisser correction for multiple comparison was applied in each repeated measures ANOVA described here.

Together, our brain results indicated that a brief training of nonsymbolic approximate arithmetic was associated with brain adaptations, which were somehow specific to number processing. Indeed, the increase in P2p amplitude across training sessions may have reflected a larger recruitment of the neural networks involved in number processing, since the amplitude of P2p amplitude in the Color-Group did not change across sessions, although the participants of this Group largely succeeded in the training task. We then proceeded to look for correlations between brain and cognitive/behavioral results.

### Correlation Analysis

We focused our correlation analyses on the relationship between standardized gains scores in the cognitive tasks, and the “gain” in P2p mean amplitude, computed as the subtraction of the P2p amplitude obtained in session 1 from the amplitude observed in session 7 (see Hyde et al., 2016 for a similar approach of using differences in the brain response, although in their case between two experimental conditions). We hypothesized that if P2p is a brain signature of ANS processes, reflecting the activity of brain regions associated with the processing of nonsymbolic and symbolic numbers, then we may find correlations between the gains in symbolic arithmetic skills and the changes in P2p.

**Figure 6:**
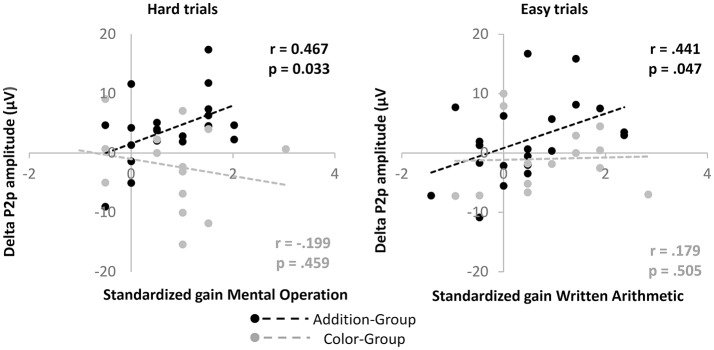
We draw the scatterplot for gain in P2p mean amplitude over the parietal clusters for the Hard and Easy trials against the standardized gain in Mental Operation and Written Arithmetic, respectively. R and *p*-values for each Group are drawn.

Indeed, we found that in the Addition-Group only, the gain in P2p amplitude for the Hard trials positively correlated with the standardized gains in Mental Operations (Person’s R coefficient = 0.466, *p* = 0.033), and that for the Easy trial positively correlated with the standardized gains in Written Arithmetic (Pearson’s R coefficient = 0.436, *p* = 0.047; Figure 6). Consistent with these results, the multiple regression analysis revealed that, only in the Addition-Group, the gain in P2p amplitude for the Hard trials was a significant predictive variable for the standardized gain in Mental Operations (Beta = 0.466, *t* = 2.990, *p* = 0.033). Although being non-significant, we also found that the P2p amplitude for the Easy trials predicted the Written Arithmetic in this Group (Beta = 0.375, *t* = 1.762, *p* = 0.094). We did not find any significant correlation concerning the Color-Group (*p* > 0.456 for each comparison).

These results suggested that the covariance between the gain in math skills and the gain in the P2p amplitude across the training in Addition-Group might reveal the two faces of a single process, that is, the training of the activation of neural networks involved in nonsymbolic and symbolic numeric processing in young children.

## Discussion

The major findings of our study showed that the improvements in a nonsymbolic approximate arithmetic training in 1st grade children are related to functional changes in brain activity, and that such changes were associated with the gains observed in symbolic arithmetic tasks after training.

Consistent with previous training interventions, we observed that children in the Addition-Group provided responses increasingly more accurate as the training progressed, suggesting that ANS abilities are malleable through intensive experience. We also observed that children in this group displayed significant gains in a test of symbolic arithmetic (Mental Operations) when compared to a passive Control-Group, indicating some transfer effects from nonsymbolic to symbolic arithmetic skills. However, the fact that we did not observe differences in cognitive gain patterns when compared to the Color-Group, suggests that these transfer effects were nonspecific.

Based on previous evidence reported by Hyde et al. (2014), we did not expect the gains in symbolic arithmetic observed in the Color-Group (although it was not significantly different from the passive Control-Group). They found that children trained in nonsymbolic addition with dot arrays performed better than children trained in performing brightness comparison, in a subsequent symbolic exact arithmetic test. It is possible that the longer duration of our training compared to theirs (7 sessions vs. 1 session) may have been enough to enhance performance in our Color-Group. This is consistent with studies that have shown cognitive interference effects between number and brightness processing, and with the overlapped activations described over the parietal cortex when solving both types of tasks (Cohen-Kadosh et al., 2008; but see Pinel et al., 2001). More generally, this result fits with the notion of a generalized magnitude system, according to which all magnitudes (numeric and non-numeric) are processed on a single mental scale and are expected to interface similarly with symbolic math abilities (Walsh, 2003; Lourenco et al., 2012; see Lourenco, 2016 for a review). However, our brain results challenge this interpretation, as they revealed marked differences between Groups precisely in a brain signature associated with magnitude comparison (the P2p component).

In fact, only the Addition-Group exhibited an increase in the amplitude of P2p, despite that both Groups exposure to identical stimuli, suggesting that both Groups exercised different mental computations during the training. In particular, since the P2p component linkage to the processing of numerical information in different experimental settings (e.g., Hyde et al., 2016; Szücs and Soltész, 2008), the specific increase of this variable in the Addition-Group may reflect that numeric brain regions became more sensitive to the processing of numerical information across the training. Note that this effect cannot be explained by differences in the difficulty of the tasks, since we observed it in both Hard and Easy trials, the latter exhibiting similar behavioral performance between Groups.

Moreover, the fact that we observed a correlation between the increase in P2p amplitude and the gains in symbolic arithmetic tasks only for the Addition-Group, not only does reinforce the idea that this ERP component indexes numerical-related processes, but further suggests differences between both training programs. Indeed, the enhancements in symbolic arithmetic skills for the Color-Group could relies on different mechanisms than those attributed to the improvement in math skills in the Addition-Group. Although we did not directly measure working memory abilities in this study, the color task may have exercised it in every trial, by requesting to keep the color intensity of the three consecutive stimuli in mind to provide the correct response. The practicing of this memory effort may be transferable in some ways to math skills. Indeed, even very simple tasks, such as color comparison, impose a memory load as they require simultaneous and sequential processes of perceiving, coding, interpreting, and comparing information (e.g., Geary, 2011; Kolkman et al., 2013). Alternatively, as this Group did not show differences from the passive Control-Group, the increase in symbolic arithmetic tests may simply reflect learning experiences with numbers at their school. It is important to notice that the lack of a significant gain in the training accuracy across sessions in the Color-Group might not prevent the transfer to math skills. Previous studies have pointed out (e.g., Park and Brannon, 2016) the fact that a training group does not show improvements in a training task, does not preclude the possibility of transfer effects to a target task (because the relevant connection would not be between the observable performance in the training and target tasks, but rather between the unobservable cognitive elements underlying the training and target tasks). Finally, since every numeric task is to some extent influenced by the magnitude aspects of the stimuli (e.g., Soltész and Szücs, 2014), further design in the current color task, such as using different levels of difficulty, would be necessary to precise better the brain response associated with the color training task.

As mentioned in the “Introduction” section, besides examining the brain correlates of nonsymbolic arithmetic training, we sought to obtain a wider picture of its potential transfer effects to symbolic math abilities. As aforementioned above, previous studies have focused on assessing transfer to symbolic arithmetic abilities, and that was the core of our math evaluations. However, we also included the Number Line task (see also Khanum et al., 2016) and a Numeration test as complementary tasks. Although we did observe gain in Number Line for the Addition-Group, this gain was similar to the one exhibited by the Control-Group, showing that this spatial number task was not sensitive to the nonsymbolic arithmetic training.

Concerning the Numeration task, the lack of gains suggests that, at least after applying our short intervention, the practice of ANS-computations may not be related to basic conceptual knowledge of numbers. This is consistent with a recent ERP study (Hyde et al., 2016), which shows that a brain signature associated with the processing of small nonsymbolic numbers (the early N1 component), but not with the ANS (P2p), was related to counting proficiency with preschool children after controlling for general cognitive factors.

While the increase in P2p amplitude for the Addition-Group across training suggested that parietal networks became more sensitive to nonsymbolic numerical magnitudes, it is reasonable to suspect that these brain changes were specifically targeted to the IPS, since previous studies described this region as a cortical generator of the P2p (e.g., Hyde and Spelke, 2012). The malleability of the ANS by training could thus have had its cortical origins in changes in the IPS activity. Indeed, the IPS has been consistently related to numerical processing in infants, children and adults (Izard et al., 2008; Dehaene and Brannon, 2011), in multiple tasks using both nonsymbolic and symbolic numbers. Our observation of a positive correlation between the gain in P2p amplitude and the gains in symbolic arithmetic for the Addition-Group is consistent with this function of the IPS of processing numerical magnitudes in general (nonsymbolic and symbolic). Moreover, our results tentatively suggest that the improvements observed in symbolic arithmetic may be caused by the changes in IPS activation (under the assumption that the P2p is an index of that activity). This possibility fits well with the proposal which states that what drives transfer between nonsymbolic and symbolic number abilities is an overlapping of brain and cognitive structures (the Representational Overlap Hypothesis, see Hyde et al., 2016). However, a couple of concerns warrant caution on this interpretation. First, although P2p can be associated with IPS activity, the changes in the amplitude of this component may be a down-stream effect of changes occurring in other brain structures during the execution of the task. In this sense, it has been shown a tight association between mental arithmetic and the processing of spatial information, pointing to the Superior Parietal Lobule as a region linking both of them, which can be dissociated from the IPS (Knops et al., 2009). Under this scenario, the transfer effects could be explained by shared mental operations between nonsymbolic and symbolic arithmetic (i.e., manipulation and transformation of numerical representations), rather than shared mental representations of numerical information *per se*. This would be in line with the Operational Overlap Hypothesis (Hyde et al., 2016). Second, we observed a lack of gains in Weber fraction in the nonsymbolic number comparison task (Halberda et al., 2008), which was somehow unexpected, although other studies have reported similar absence (e.g., Hyde et al., 2014; Khanum et al., 2016). This negative result suggests that neither the improvements in the nonsymbolic arithmetic task nor the potential transfer effects to symbolic math were associated with fundamental changes in the ANS acuity. Thus, if the Representational Overlap account is valid, then the transfer effects may not involve the sharpening of tuning curves in IPS neurons, supposedly coding both symbolic and nonsymbolic numerical magnitudes (Dehaene and Brannon, 2011) as previously suggested (Park and Brannon, 2013). Further studies are certainly necessary to decide between these possibilities and specify in more detail the brain mechanisms underlying ANS and symbolic math connections.

Finally, in a more applied vein, the weak transfer effects we observed here, along with other recent reports, suggest caution when considering our results as inputs to potential applications of ANS training interventions to math education. Indeed, a recent large-scale longitudinal experiment reported weak long-term effects of an ANS-based training intervention (Dillon et al., 2017). These authors observed that while practicing ANS games with preschool children led to enduring positive effects in nonsymbolic number abilities compared to a Group trained in social games (measured up to around 1 year later), only weak and short-lived effects were seen in symbolic math skills after the intervention. As these and other authors have suggested (Lewis et al., 2015), it is possible that stronger benefits in symbolic math skills may be attainable if these training paradigms could exercise simultaneously intuitive and formal aspects of math knowledge. Notwithstanding, more experimental designs, such as those we and other previous studies have employed (e.g., Hyde et al., 2014), are necessary to better test and elucidate eventual causal relationships between symbolic and nonsymbolic abilities, which may serve as inputs to educational purposes. Additionally, how to properly use and take the best advantage of computer technologies in teaching math (or any subject) is something that also warrants further research (Geary et al., 2008).

## Conclusion

Our study provides a new piece of neurocognitive evidence on the links between intuitive and formal math abilities, showing that a multi-session ANS arithmetic training led to behavioral and brain changes in number specific networks in young children. We believe that our data would serve as an input for current models aimed at characterizing math cognition during early development.

## Author Contributions

CG and MP designed research, analyzed data and wrote the manuscript. CG, CGS, BG and MP performed research. BG edited the manuscript.

## Conflict of Interest Statement

The authors declare that the research was conducted in the absence of any commercial or financial relationships that could be construed as a potential conflict of interest.
